# A new bifunctional hybrid nanostructure as an active platform for photothermal therapy and MR imaging

**DOI:** 10.1038/srep27847

**Published:** 2016-06-14

**Authors:** Mona Khafaji, Manouchehr Vossoughi, M. Reza Hormozi-Nezhad, Rassoul Dinarvand, Felix Börrnert, Azam Irajizad

**Affiliations:** 1 Institute for Nanoscience and Nanotechnology, Sharif University of Technology, Tehran 14588, Iran; 2 Department of Chemical and Petroleum Engineering, Sharif University of Technology, Tehran, Iran; 3 Institute for Biotechnology and Environment (IBE), Sharif University of Technology, Tehran, Iran; 4 Department of Chemistry, Sharif University of Technology, Tehran 11155-9516, Iran; 5 Nanotechnology Research Center, Faculty of Pharmacy, Tehran University of Medical Sciences, Tehran, Iran; 6 IFW Dresden, PF 270116, 01171 Dresden, Germany; 7 Speziallabor Triebenberg, TU Dresden, 01062 Dresden, Germany; 8 Department of Physics, Sharif University of Technology, Tehran 14588, Iran

## Abstract

As a bi-functional cancer treatment agent, a new hybrid nanostructure is presented which can be used for photothermal therapy by exposure to one order of magnitude lower laser powers compared to similar nanostructures in addition to substantial enhancment in magnetic resonance imaging (MRI) contrast. This gold-iron oxide hybrid nanostructure (GIHN) is synthesized by a cost-effective and high yield water-based approach. The GIHN is sheilded by PEG. Therefore, it shows high hemo and biocompatibility and more than six month stability. Alongside earlier nanostructures, the heat generation rate of GIHN is compareable with surfactnat-capped gold nanorods (GNRs). Two reasons are behind this enhancement: Firstly the distance between GNRs and SPIONs is adjusted in a way that the surface plasmon resonance of the new nanostructure is similar to bare GNRs and secondly the fraction of GNRs is raised in the hybrid nanostructure. GIHN is then applied as a photothermal agent using laser irradiation with power as low as 0.5 W.cm^−2^ and only 32% of human breast adenocarcinoma cells could survive. The GIHN also acts as a dose-dependent transvers relaxation time (T_2_) MRI contrast agent. The results show that the GINH can be considered as a good candidate for multimodal photothermal therapy and MRI.

Size and shape-dependent properties of metallic nanoparticles has been the subject of abundant international researches^1^,^2^,^3^. Iron oxide and gold nanoparticles have been attracted more attention compared to other metallic nanoparticles because of their special magnetic and optical properties^4^,^5^,^6^,^7^,^8^. These nanoparticles provide notable potential for a wide range of applications^9^,^10^,^11^.

The plasmonic shape-dependent properties of gold nanostructures including spherical nanoparticles^4^,^5^, nanorods^9^,^10^,^11^,^12^,^13^,^14^,^15^,^16^,^17^, nanoshells^6^ and nanocages^18^ make them suitable candidates for sensing^9^, biological^4^,^5^,^9^,^10^,^11^,^12^,^13^,^14^,^15^,^16^,^17^ and optoelectronic^15^ applications. Anisotropic gold nanorods (GNRs) are an important member of noble-metal particles because of their unique advantages compared to other structures. Some of these advantages are tunable longitudinal surface plasmon resonance (LSPR) throughout visible and near IR region, enhanced absorption and Rayleigh scattering per volume unit and high sensitivity against changes in local environments, in addition to ease of synthesis and biocompatibility and improved cellular uptake^15^,^16^,^19^,^20^.

Alongside these advantages, there are some drawbacks in utilizing GNRs especially in biological applications. The surfactant-directed process is the most popular GNRs synthesis method^10^,^11^,^19^,^21^,^23^. Beside its advantages, presence of free and surface desorbed surfactant molecules in final product, make significant cytotoxicity to all cell-lines^13^,^14^,^16^,^17^. These surfactant molecules should be removed or passivated before the usage of GNRs^13^,^14^,^17^. The other issue is the lack of GNRs stability. By removing cetyl trimethylammonium bromide (CTAB) as a capping agent, GNRs are aggregated and undergo to below melting point reshaping^1^,^14^,^16^,^20^. Furthermore, during the routine process of centrifugation for omission of excess CTAB, GNRs reformed to bulk materials after third step of washing CTAB^12^.

There are some modification methods to improve the stability and biocompatibility of GNRs. “PEGylation” is the current technique to solve some of these problems. Polyethylene glycol (PEG) molecules enhance the biocompatibility of GNRs by preventing protein adsorption and GNRs aggregation^13^,^14^,^16^,^17^,^19^. Also, a PEG layer decreases the surface diffusion which results in deceleration in GNRs reshaping^1^. But there are some difficulties in this modification: firstly, only thiol-terminated polyethylene glycol can be directly attached to the surface of GNRs^15^,^16^,^23^,^24^ and secondly, even by this modification, centrifuge-based washing steps cannot be omitted^19^,^20^. So, biological use of PEG-modified GNRs is not cost-effective, requires special reagent and multi-steps low-yield treatments. These unresolved problems, limit the commercial applications of GNRs.

Another method to improve GNRs bio-ability which some researches have recently attempted for, is the synthesis of novel hybrid nanostructures^5^,^6^,^7^,^11^,^17^. Among other nanoparticles for synthesis of hybrid structures with GNRs, superparamagnetic iron oxide nanoparticles (SPIONs) are good candidates because of their biocompatibility^7^,^8^,^25^,^26^,^27^,^28^,^29^,^30^,^31^. Combining nanoparticles with surface plasmon resonance properties and superparamagnetic properties into one single structure can lead to a development of new functional and efficient techniques in therapeutic^24^,^32^,^33^, bioimaging^21^,^33^,^34^ and biosensing^2^,^35^. Such hybrid nanostructures are divided into two types, core/shells and hetrodimers. While the SPION/gold core/shell nanostructure has been more frequently used because of its advantages in hyperthermia therapy^6^,^33^,^36^,^37^, it has some drawbacks which should be taken into consideration. The decrease in superparamagnetic properties of SPIONs due to the diamagnetic nature of gold shell^34^ and the damping in surface plasmon resonance peak intensity caused by complex refractive index of SPIONs^31^ are common issues, which enforced the use of silica^34^ or polymer molecules^31^ as a shielding layer. Additionally, the majority of spherical geometry of SPION/gold core/shell nanostructures just leads to one plasmon resonance mode, which is less sensitive to changes in the dielectric properties of the surrounding, compared to the longitudinal ones^38^. On the other hand, heterodimers can be a promising nanostructure as they benefit from the advantages of both single nanostructures and do not show the inherent weakness of a core/shell structure.

As far as we know, there are only few reports about the synthesis of a gold nanorod-SPIONs heterodimer nanostructure^22^,^23^,^24^,^38^. Electrostatic intractions^22^,^38^, hetrofunctional molecules^22^,^23^ and amid groups formation^24^ have been used for the attachment of two nanostructures. In majority of these reports, a noticeable decrease in the LSPR peak intensity could be observed^22^,^23^,^38^. The cause of this damping is that the distance between the magnetic and plasmonic structures is not large enough. Hence, the nanostructures cannot behave as they are separated^39^. In this work, we report a simple, cost-effective, and high-yield procedure to synthesize a stable, yet magneto-plasmonic nanostructure. To the best of our knowledge, this is the first time that the distance between two nanostructures is adjusted in a way that the maximum plasmonic absorption of GNRs could be obtained. This enhancement in the LSPR led to an increase in the laser absorption. In addition to this, high fractions of GNRs in the final nanostructure increase its ability for photothermal therapy. The mentioned characteristics allowed utilizing lower power levels of the laser. The SPION to GNRs ratio and the percentage of PEG as stabilizer agent are also optimized for enhanced MRI negative contrast and at the same time for an improved photothermal effect. Because of magnetic properties of the GIHN, complete removal of toxic CTAB surfactant without damaging the GNRs became possible. The biocompatibility, hemocompatibility, photothermal and MRI ability of the final structure is examined as well. Based on the results, this hybrid nanostructure can be used for simultaneous photothermal therapy and contrast enhanced MRI.

## Result and Discussion

### Synthesis of gold nanorods (GNRs)

GNRs were synthesized by a seed-mediated method. Similar to older reports^3^, every change in the amount of each reagent in growth solution led to changes in GNR’s size and aspect ratio. In order to control the GNR’s aspect ratio, only the silver nitrate amounts were changed. The amounts of all the other reagents were kept constant. An aliquant of 50 to 60 μl of 0.01 M AgNO_3_ was used to obtain rods with aspect ratio of 4. Additionally, the results show that the synthesis temperature is an important parameter to provide majorly monodispersed rods with a very low amount of byproducts. Therefore, all of the synthesis steps were done at a constant temperature of 30 °C.

**Figure 1:**
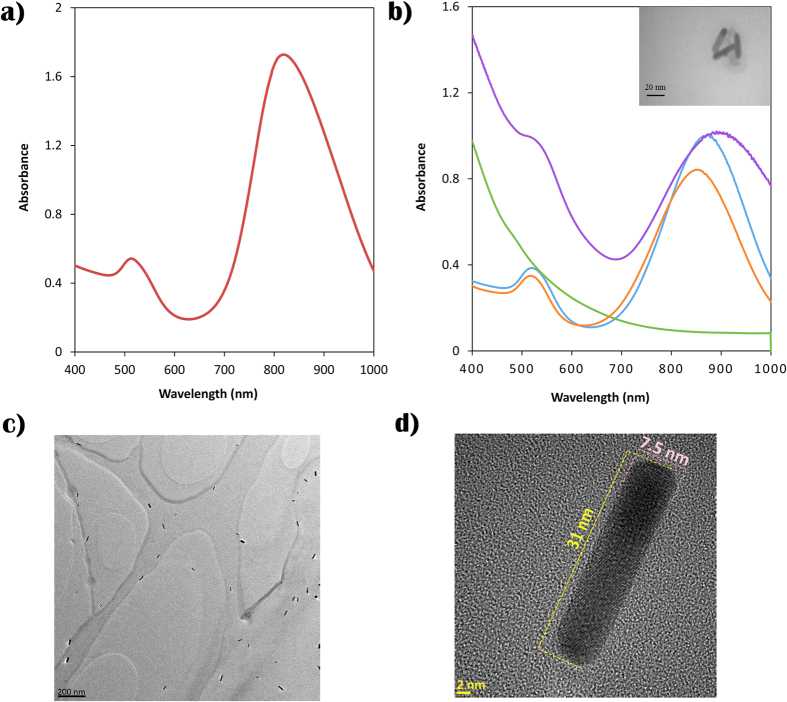
UV-Vis absorption spectra of synthesized gold nanorods (**a**), UV-Vis spectra of SPIONs (green line), GNRs (Blue line), cystamine modified GNRs (red line) and GIHN (violet line). (**c**) TEM image of GIHN (inset c), TEM image of GNRs (scale bar is 200 nm) (**c**), HR-TEM image of GNR (scale bar is 2 nm).

The UV-Vis spectrum of synthesized GNRs is presented in Fig. 1a. It can be seen that a symmetric longitudinal surface plasmon resonance peak of the rods appears in the near IR region. The LSPR peak position is affected by the aspect ratio as shown in the following equation:^40^


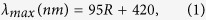


where R is the GNR’s aspect ratio. According to Equation 1 the aspect ratio of the synthesized GNRs was calculated to be almost 4.18. Also the HR-TEM images in Fig. 1c,d show that the synthesized GNRs had an almost monodispersed aspect ratio of 4 with a narrow size distribution (Supplementary Figure S1). The GNR’s length is less than 32 nm and their width does not reach 8 nm.

### Synthesis of GIHN

The GNRs were synthesized by seed mediated method in 0.1 M CTAB. This nearly CTAB-saturated media limits a lot of reaction progresses. Thus, it is necessary to remove CTAB or decrease its concentration before any modification or usage of GNRs in biological and sensing applications.

The common method utilized for this purpose is washing the GNRs through centrifugation. But as reported before by J. Manson *et al*., any centrifuge step causes a degree of damage to GNRs^20^. Therefore, it would be better to choose a modification process like self-assembly which is done in a concentrated CTAB media^23^,^24^. As is known, sulfur groups can be self-assembled on a gold surface. So, sulfur containing molecules could be used for modification of GNRs with no need to pretreatment. On the other hand, in order to make the attachment of GNRs and SPIONs possible, a thiol functionalized linker with primary amine groups was needed. Cystamine, an organic disulfide diamine, was used in this case which can be self-assembled to the gold surface using the sulfur groups and the terminated amine groups were remained untouched to react with carboxylic groups of the SPIONs. The surface plasmon resonance of the GNRs is very sensitive to changes in the local environment^19^, so the successful replacement of cystamine molecules with the CTAB ones was checked by detecting the changes in λ_SPR_. A UV-Vis absorption spectrum is presented in Supplementary Figure S2. It shows a 24 nm blue shift in λ_SPR_ which occurred because of the deference in refractive index of cystamine compared to CTAB.

The amine modified GNRs have an electrostatic attraction to the carboxyl functionalized SPIONs. However, the formation of a covalent amid binding was achieved by the addition of EDC and NHS reagents. There are several parameters affecting the quality of the final product and they should be optimized.

#### SPION to GNR ratio

The PEG-modified SPIONs amount should be optimized so that the minimum numbers of SPIONs remain free after the reaction and the maximum numbers of GNRs are attached to the SPIONs. Additionally, the product should demonstrate both plasmonic and magnetic properties and the least amount of aggregation should have been occurred.

To investigate the effect of SPIONs ratio different amounts of PEG modified SPIONs suspension were added to various portions of the same amine functionalized GNRs solution under stirring. The product and supernatant of each one were analyzed at the end of the reaction. At first, low ratios (5 to 15%w/w) of SPIONs to GNRs were added and the reaction was allowed to proceed. The analysis shows that the supernatant contained free GNRs after magnetic separation but the amount of free GNRs decreased with increasing the SPION’s ratio. This means that the ratio of SPIONs was not sufficient to be attached to all GNRs. For ratios of 20 to 25%w/w the amount of gold in the supernatant was negligible. By raising the ratio of SPIONs to 35%w/w, at first a dose-dependent aggregation was observed. For higher ratios though, there were free SPIONs which could be separated through centrifugation. So the ratio of 20% w/w was selected as an optimum amount in this synthesis.

#### Distance between two nanostructures

As it was described before, the distance between two nanostructures affects their properties. When iron oxide nanoparticles were synthesized *in situ* on the surface of GNRs, the plasmonic resonance peak completely disappeared because the gold surface was not available anymore^22^. The intensity of the LSPR peak was reduced to the half compared to bare GNRs using the HS-PEG 5 kDa linker^23^. As a result of the complex refractive index of Fe_3_O_4_, SPIONs are able to absorb a portion of the GNR’s surface plasmon and decrease the LSPR peak intensity^31^. Therefore, the effect of the absorption due to surface plasmon became more important by increasing the dielectric constant of the non-plasmonic component of the structure^39^.

In order to investigate the effect of the distance between both structures, two different PEGs were used for SPION modification, 6 kDa and 10 kDa. As could be seen in Supplementary Figure S3, when 6 kDa PEG-diacid was used as a linker, the LSPR peak intensity considerably decreased. However, when 10 kDa PEG-diacid was used for SPION modification, the distance between GNRs and non-plasmonic iron oxide nanoparticles became long enough and the SPR absorption by the SPIONs was negligible. Thus, the SPIONs modified by PEG 10 kDa were used in synthesis of the plasmo-magnetic structure.

#### GIHN stabilizing

By continuing the amide formation reaction between NH_2_-modified GNRs and carboxylated SPIONs in presence of EDC and NHS, a highly aggregated product was achieved.

So, the reaction should be quenched by an addition of another reagent which could prevent nanoparticles aggregation.

One hour (60 min) after starting the reaction, almost all of the GNRs could be collected from the suspension using an external magnetic field. This shows that the reaction was acceptably progressed. Therefore, after an hour from the addition of the SPIONs, carboxyl functionalized PEG was added to the mixture as a reaction quencher and stabilizing agent. Polyethylene glycol is a good candidate for nanostructure stabilization because of its biocompatibility. It could react with free amine groups on the surface of GNRs.

The weight percentage and the length of PEG chain can influence the product quality. For this purpose, the suspension stability, LSPR peak intensity compared with CTAB-capped GNRs, and redispersion after freeze drying were studied. Different amounts of either diacid-PEG 6 kDa or 10 kDa (2 to 5-fold mole carboxylic/mole amine) were examined for stabilizing the GIHN, but none of them results to a stable product. The best results were obtained when a mixture containing 5-fold mole carboxylic/mole amine of mPEG-acid 6 kDa and diacid-PEG 10 kDa (2:1) was used as a stabilizing agent. The resulting suspension was stable for at least one week at room temperature and after that could be completely redispersed after 30 seconds of sonication. The LSPR peak intensity and position did not show a significant change when compared to unmodified GNRs even after a drying and redispersing step. Therefore a 5-fold mole carboxylic/mole amine mixture of 6 kDa mPEG-carboxylic acid and 10 kDa dicarboxylated-PEG was selected as an optimized amount for stabilizing the GIHN.

To investigate the reproducibility of the method, the GIHN was synthesized ten times according to the optimized conditions mentioned previously. The results were almost the same as long as the optical density of GNRs solutions were similar and their aspect ratios remained between 3.5 and 4.5.

Due to the SPION’s connection, not only the intensity of LSPR peak was affected but also its position was shifted. Because the refractive index of the surrounding medium was increased compared to the previous step, the addition of cystamine, red shift in the LSPR was expected. In addition, the base line of absorption was changed and reshaped to that of SPIONs after the attachment of nanostructure^38^,^39^. As shown in Fig. 1b change in the absorption base line and the LSPR red shift confirmed the formation of the hybrid structure.

**Figure 2:**
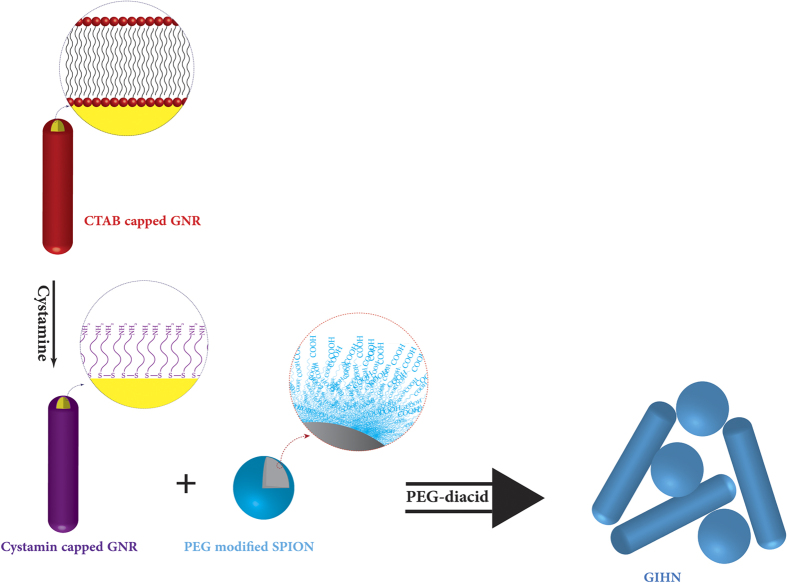
Scheme of the synthesis progress.

TEM images also show that GIHN were successfully synthesized. As it can be seen in Fig. 1b (inset) every 1–3 GNRs are attached to several SPIONs and all of them are surrounded by a PEG layer which separated this collection from the others and protected the hybrid structure from reshaping and damaging by sonication or other external tensions. The final size of the GIHN was less than 70 nm which makes it suitable for biological applications. The process is shown schematically in Fig. 2.

### Nanostructure stability

Since the final goal of GIHN synthesis is biological application, its stability during time is an important parameter which highly affects the nanostructure quality. This property was checked by monitoring the plasmonic peak changes using UV-Vis spectroscopy. For the nanostructure suspension the UV-Vis spectra did not show significant fluctuations in the LSPR absorption even after 180 days from the synthesis time when the product was kept at room temperature (Supplementary Figure S4a). As could be seen in Supplementary Figure S4b, CTAB capped GNRs solution was not as stable as GIHN in the same conditions and its LSPR peak was broadened and shifted to shorter wavelengths only after 60 days.

In addition to time stability, the GIHN could bear ultrasound waves for 7 minutes. There was no change in the LSPR peak position even after 7 minutes continuous sonication (Supplementary Figure S5). Therefore, modification of GNRs by SPIONs and PEG enhances their time stability as well as their tension bearing.

### Ability for biological application

The synthesized GIHN was aimed for photothermal therapy application, so its biocompatibility and heat generation should be examined.

#### Biocompatibility

As mentioned before, surfactant capped GNRs are very toxic because the CTAB molecules could be desorbed from the rod’s surface and cause cell death^13^,^14^,^16^,^17^. Therefore GNRs could not be used in biological systems before completely replacing the toxic CTAB molecules with a biocompatible stabilizer like thiol-functionalized PEG^13^,^14^,^16^,^17^,^19^. PEGylation is the most efficient antiopsonization strategy. The linear PEG molecules inhibit the protein adsorption as a result of steric hindrance. Therefore, the immune system cannot recognize the nanoparticles as foreign spices^41^. The synthesized GIHN was shielded by PEG so it was expected that the biocompatibility of this structure would be acceptable.

**Figure 3:**
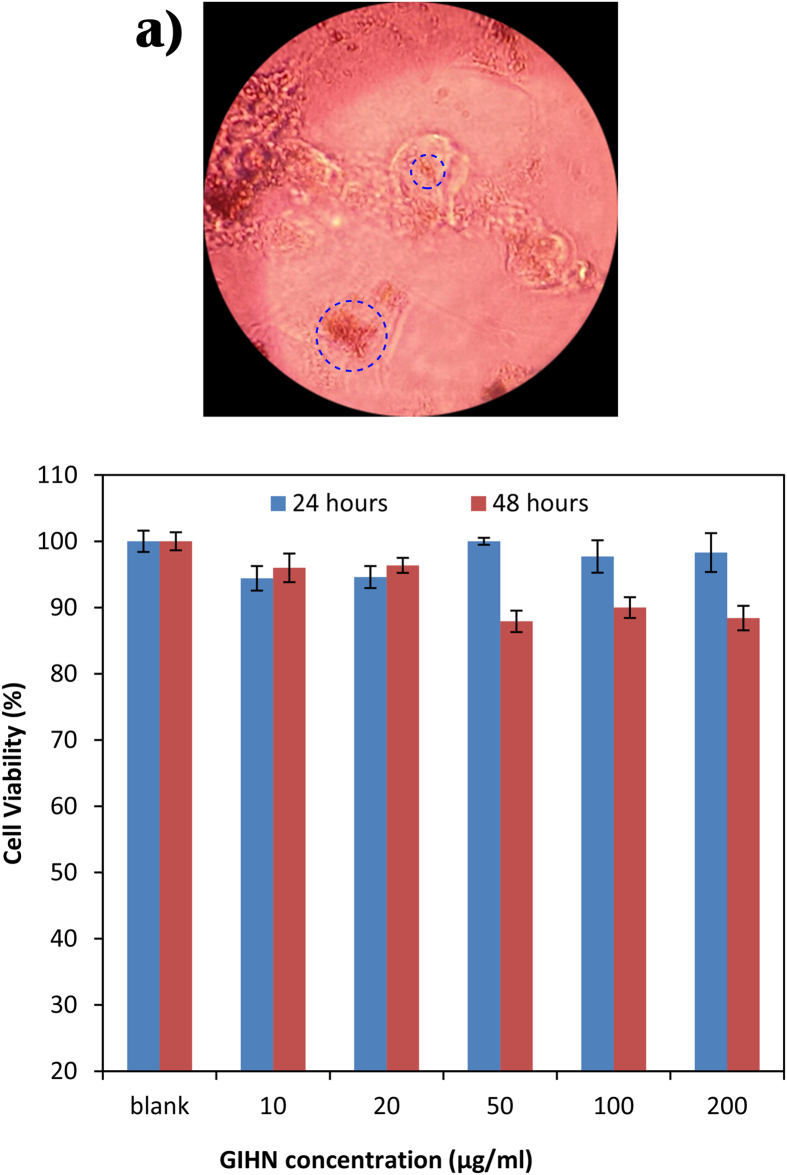
Transmission light microscope image of cellular uptake of GIHN (shown by blue circles) (**a**), L929 cell viability after 24 and 48 hours incubation with different concentration of GIHN (**b**).

The biocompatibility of the new nanostructure was examined by the MTT assay test. For this purpose, different concentrations of nanostructure suspension with the maximum of 200 μg/ml were provided in an RPMI medium which contains 10% V/V of FBS. L929 cells which were at their exponential phase of growth curve were incubated with nanostructures for 24 and 48 hours. The results show that these particles had no remarkable cytotoxicity and more than 85% of cells remained alive even at high concentrations of GIHNs compared to the blank sample. Figure 3a,b show the cellular uptake of nanoparticles and the cell viability bar chart after 24 and 48 hours of incubation at different concentrations, respectively.

#### Hemocompatibility

Any foreign biomaterial which directly or indirectly makes a contact with blood tissues would interact with blood cells. The hemocompatibility of the new synthesized nanostructure was investigated by studying of blood coagulation and the morphology of human red blood cells (RBCs).

The blood coagulation would happen through three pathways; intrinsic, extrinsic and common pathway and is measured by the prothrombin time (PT) and activated partial thromboplastin time (APTT). The time required for a fibrin clot to form after adding tissue thromboplastin is called PT; while APTT is the time needed for a fibrin clot to be formed after the clotting process is initiated by addition of thromboplastin reagent and CaCl_2_. Biomaterials usually induce intrinsic coagulation which could be measured by APTT. To evaluate the performance of the extrinsic coagulation pathway, PT can be measured^42^.

The plasma coagulation in presence of 200 μg ml^−1^ of GIHN was investigated. The results show that the APTT and PT values in presence of GIHN were remained in the normal range when compared to the phosphate buffer saline (PBS) control. So the plasma coagulation was not affected by even such high concentrations of synthesized nanostructure.

**Figure 4:**
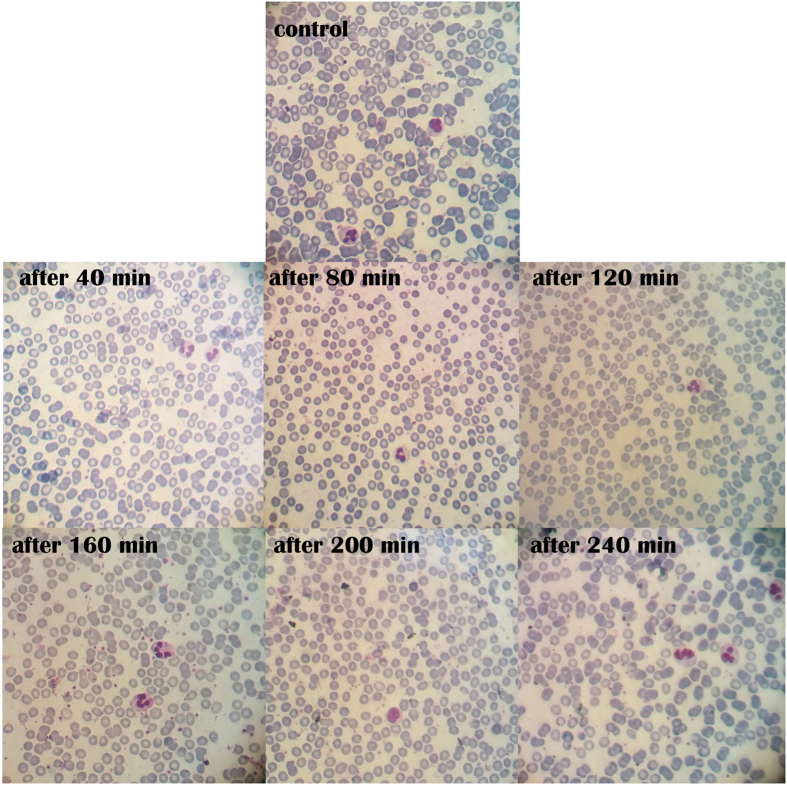
Microscopic pictures of blood smears prepared from EDTA-anticoagulated blood without GIHN (control) and after addition of 200 μg ml^−1^ of GIHN in 40 min intervals up to 240 minute.

Morphology of the RBCs is the important parameter which is strongly impacted by the interactions between red blood cells and biomaterials. Normally RBCs have a biconcave disk shape and their surface is negatively charged, so electrostatic interaction between polymers and RBCs easily affects their shape^42^. Peripheral blood smear test was performed to study the influence of GIHN on the shape of RBCs. Red blood cells morphology was observed after exposure to 200 μg ml^−1^ of GIHN for up to 4 hours. Figure 4 shows that RBCs are undergone neither deformation nor aggregation after different time periods of exposure to nanostructure comparing to the control. These results show that the synthesized GIHN is displaying excellent hemocompatibility. This excellent hemocompatibility was resulted from the PEG coating which prevented the protein attachment as discussed earlier. Also the almost natural surface of GIHN minimized the interaction between the nanostructure and blood cells leading to enhanced hemocompatibility.

#### Photothermal effect

**Figure 5:**
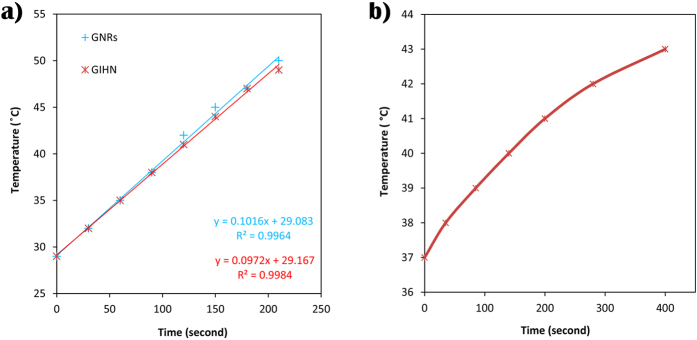
Rate of heat generation by laser absorption of GNRs (blue line) and GIHN (red line) (**a**), rate of heat generation by laser absorption of DMED medium containing 200 μg/ml of GIHN (**b**).

The ability of GIHNs for heat generation caused by laser beam absorption was evaluated and compared with surfactant-capped GNRs. The results were almost the same when the concentration of gold in two samples was 0.1 mg in 1 ml of DI water and exposed to a laser beam at a wavelength of 808 nm with a power of 0.5 Wcm^−2^. So the presence of SPIONs and shielding layer of PEG did not affect the laser absorption of GNRs. The medium temperature was increased linearly with exposure time for both of GNRs and GIHN suspensions. Figure 5a shows the lines and their related equations. The temperature was recorded by k-type thermocouple versus time of exposure.

The heat generation of GIHN also was tested in DMED medium which contain 10% FBS at the concentration of 200 μg/ml. As could be seen in Fig. 5b, the temperature of suspension rose to 43 °C in 7 minutes after being exposed to laser irradiation with a power of 0.5 W/cm^2^ where the total volume of sample was 1 ml. Because of high fraction of GNRs in the GIHN and excellent LSPR, laser irradiation with one order of magnitude lower power levels than the previous report could be applied^23^,^43^. These results show that the GIHN can be utilized in phtothermal therapy.

**Figure 6:**
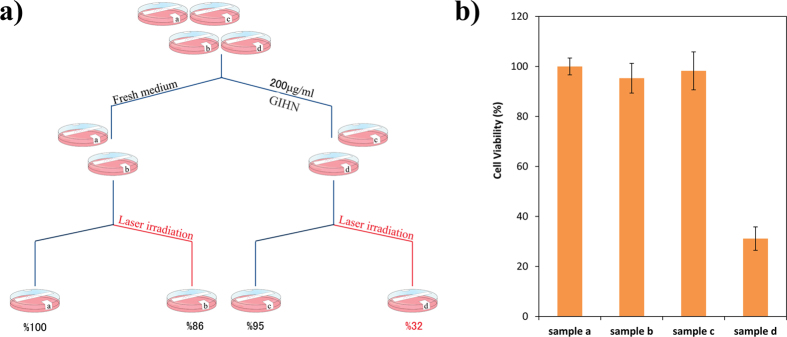
Schematic of cell treatments (**a**), cell viability of MCF7 cell after irradiation by 0.5 W/cm^2^ near IR laser (sample b), incubation with 200 μg/ml of GIHN (sample c) and incubation with nanoparticles and laser irradiation (sample d) comparing to blank (sample a) (**b**).

*In vitro* tests of hyperthermia therapy were carried out by culturing the human breast adenocarcinoma cell line (MCF7) in four samples to investigate the effect of laser irradiation, nanoparticles, and photothermal therapy one by one. The MCF7 cells were cultured in 24-well plate for 24 hours. As illustrated in Fig. 6a, the samples were divided in two subgroups. For one of them the culture medium was replaced with the fresh DMEM medium (samples a and b). In the other subgroup, the culture medium was replaced with suspension of 200 μg/ml GIHN in DMEM (samples c and d). After additional 24 hours of incubation, one sample from each subgroup was exposed to an expanded laser beam at a wavelength of 808 nm (0.5 W/cm^2^) for 7 minutes. All the groups were incubated once more for 24 hours, and then the standard MTT test was used for evaluation of cell viability. Results showed neither laser beam nor nanostructure could cause remarkable cell death. But when the GIHN was used as a photothermal agent, an effective decrease of 68% could be observed in cell viability. Figure 6b shows the average of three replications.

In addition to the higher fraction of GNRs in the GIHN, cellular uptake was the other parameter behind of the efficient photothermal treatment. The cellular uptake itself is directly related to the interaction between nanoparticles and cell membrane. This interaction is affected by nanostructure’s morphology, size and surface properties. Earlier studies showed that nanostructures with spherical morphology internalized faster than other shapes^41^,^44^,^45^. Regarding the size of the nanostructure, particles between 50 to 100 nm are proved to have higher internalization^45^. Furthermore the most important parameter is surface properties^44^. Replacement of surface adsorbed toxic molecules like as CTAB with PEG is a popular strategy for increasing the cellular uptake^44^.

As a result of total replacement of CTAB with PEG molecules and average size of 70 nm in this work, it was expected that the GIHN could internalize and aggregate into the cells causing an efficient photothermal effect after exposure to laser irradiation.

#### MRI contrast effect

**Figure 7:**
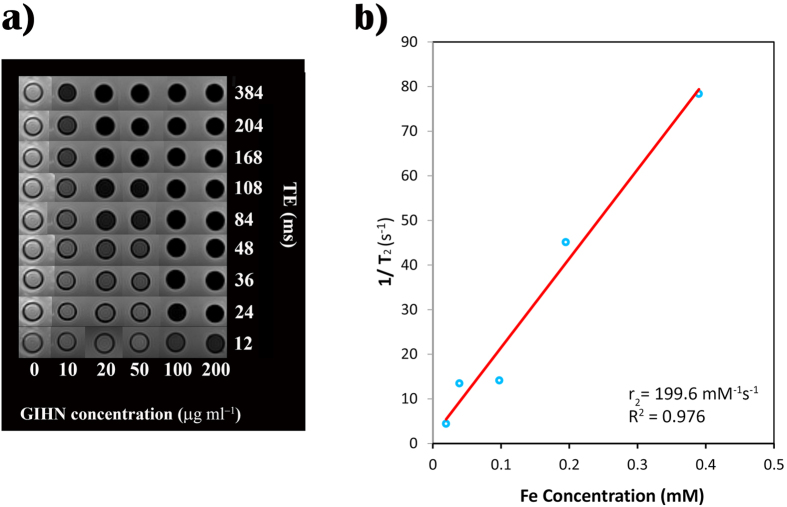
T_2_-weighted MR images of GIHN in aqueous media at various concentrations of iron and different echo times (**a**). T_2_ relaxation rate (R_2_) versus iron concentration due to hybrid nanoparticles (**b**).

To investigate the MRI sensitivity of GIHN as a transvers relaxation time (T_2_) contrast agent in clinical diagnosis, magnetic resonance (MR) studies were accomplished using a clinical 3 T MRI scanner. The MR images of aqueous solutions containing various amounts of GIHN (0 to 200 μg/ml) show a significant dose-dependent negative contrast enhancement compared to the deionized water control (Fig. 7a).

TE was scanned from 12 to 384 ms and MR images were recorded. The T_2_ of the water proton in different solutions was calculated by fitting a logarithmic curve to the mean of the measured MR signals. The transvers relaxivity, r_2_, which is the general measure for contrast agent ability, was calculated by linear least square fitting of 1/T_2_ (s^−1^) versus iron concentration (mM)^37^,^46^.

The obtained transvers relaxivity for the synthesized GIHN was 199.6 mM^−1^ s^−1^ (Fig. 7b) which was higher than that of the r_2_ amounts which were obtained for cluster-like structures^46^.

However the r_2_ reported amounts for the bare iron oxide nanoparticles with diameters between 15 nm and 25 nm were 60–70 mM^−1^ s^−1^, but there was a significant enhancement in transvers relaxivity for the aggregated cluster-like nanostructures^33^,^43^. There are several structural parameters which would affect the magnetic resonance images. The most important one is related to the SPIONs arrangement and attachment to each other and this caused the increase in r_2_. The SPIONs arrangement affects the magnetic behavior of the hybrid nanostructure in several ways. Firstly, the crowding of multiple iron oxide nanoparticles in the GIHN shortens the distance among magnetic particles which, in turn, leads to magnetic coupling among iron oxide nanoparticles. So the synergistic magnetic effect causes stronger magnetization for GIHN when compared to free SPIONs^24^,^46^. Secondly, as reported earlier, the transvers relaxivity is related to the square of single nanoparticle’s diameter while for the clusters, r_2_ is related to the square of hydrodynamic diameter of aggregated structure^47^. The larger hydrodynamic diameter of PEG shielded GIHN is the reason behind the enhanced transvers relaxivity and darker MR images. Finally, the hydrophilic surface of synthesized nanostructure facilitates the diffusion of water molecules near the surface of magnetic particles and extends the interaction between magnetic field and protons. This also increases the relaxivity time^46^. Because GIHN contains multi-magnetic particles in the shortened distance and is coated with hydrophilic PEG, it could serve as a highly efficient T_2_ MRI contrast agent.

## Methods

### Materials

Silver nitrate (AgNO_3_), sodium borohydride (NaBH_4_), L-ascorbic acid, iron (III) chloride hexahydrate (FeCl_3_.6H_2_O), iron (II) chloride tetrahydrate (FeCl_2_.4H_2_O), chromium trioxide (CrO_3_), polyethylene glycol (PEG, Mw~6000, Mw~10000 kDa), methoxy polyethylene glycol (m-PEG, Mw~6000 kDa), 1-ethyl-3-(3-dimethylaminopropyl) carbodiimide hydrochloride (EDC), ammonia (NH_3_, 25%), hydrochloric acid (HCl, 37%), sulfuric acid (H_2_SO_4_, 96%), acetone and isopropanol were purchased from Merck. N-hydroxysuccinimide (NHS), cystamine dihydrochloride, penicillin-streptomycin and 3-(4,5-Dimethylthiazol-2-yl)-2,5-Diphenyltetrazolium Bromide (MTT) were obtained from Sigma-Aldrich. Cetyl trimethylammonium bromide (CTAB) and methanol were purchased from Chemlab. Hydrogen tetrachloroaurate (HAuCl_4_.3H_2_O) was obtained from Alfa Aesar. Fetal bovin serum (FBS) and RPMI 1640 and high glucose DMED cell culture mediums were purchased from Gibco. All chemicals were of analytical grade and used as received without further purifications. For the preparation of all samples, deionized (DI) water with resistivity of 18.2 MΩ was used.

### Equipment for characterization

Functional groups of polyethylene glycol were recognized by Fourier Transform infrared spectrophotometer (ABB Bomem, MB-100) and proton nuclear magnetic resonance (Brucker, AC-80). The morphology of the gold nanorods and hybrid nanoparticles were observed by high-resolution transmission electron microscopy (FEI Tecnai F20 with a CEOS CETCOR, operated at 200 kV) and transmission electron microscopy (Zeiss EM10C, 80 kV). Gold concentrations were quantified using inductively-coupled plasma-optical emission spectroscopy (Spectro, Arcos). Digestion of samples was performed in aqua regia before measurements. The UV-Vis absorption spectra were recorded on a spectrophotometer (Perkin Elmer, lambda 950) with the wavelength ranging from 400 nm to 1000 nm. All the samples were irradiated by continuous wave diode laser coupled with optical fiber (Hi-Tech optoelectronic, 808 ± 5 nm). All the magnetic resonance images were recorded using a 3T MRI scanner (Magnetotrio, Siemens).

### Polyethylene glycol functionalization

PEG-dicarboxylic acid was achieved using Jone’s reagent as described by B. S. Lele with some modifications^48^. This reagent can oxidize the primary alcohols to carboxylic acid at room temperature without side reactions. Briefly, a defined amount of PEG was dissolved in heated acetone to obtain a homogenous 0.01 M solution. This solution was allowed to attain room temperature. In a single portion, an amount of Jone’s reagent containing 0.02 M CrO_3_ was added to the reaction mixture. The appearance of blue-green coloured fine suspension demonstrates chromium salt formation. This mixture was stirred at room temperature overnight (16 hours). After that, the reaction was quenched by addition of isopropanol as a free radical scavenger^48^. Efficient separation of the chromium salt as by-product could be done by use of active charcoal (10% wt. of PEG). The reaction mixture was stirred with finely powdered active charcoal for 2 hours before filtration. The clear solution was concentrated using a vacuum evaporator. The obtained viscous liquid was poured into a petri dish and was allowed to cool down to room temperature which solidified it. However, a complete separation of the chromium salt was not possible by only applying active charcoal. The resulting products would also contain excess amounts of acid. Here, in contrast to the former work^48^, the product was then dissolved in methanol and was washed through an alumina column, concentrated using a vacuum evaporator, and solidified at room temperature. The product was white flakes. Synthesized PEG-diacid was characterized by H^1^-NMR and IR spectroscopy. In the IR spectrum shown in Supplementary Figure S6a, a sharp peak appeared at 1726 cm^−1^ after functionalization which is absent in the case of PEG. This peak corresponds to the carbonyl frequency of COOH group. The H^1^-NMR spectra in Supplementary Figure S6b exhibit a peak for carbonyl protons at 8–10 ppm. A comparison of these results to previous works^41^ confirmed that oxidation of PEG was successfully happened.

### Synthesis of GNRs

GNRs were synthesized by seed-mediated surfactant-directed method which was reported by Nikoobakht and El-Sayed^49^. The aspect ratio of rods was adjusted by changing the silver nitrate amounts as reported earlier by our group^3^. In summary, the seed solution was prepared in two steps. At first, 50 μl of 25 × 10^−3^ M HAuCl_4_ was added to 5 ml of 0.1 M CTAB and then 300 to 600 μl of freshly ice-cooled 0.01 M NaBH_4_ solution was added to the mixture under vigorous stirring. As a result, a brownish yellow solution was formed. This seed solution was stirred for 10 minutes and then stand at room temperature for 2 hours before use.

The growth solution was prepared by mixing 5 ml of 0.1 M CTAB, 100 μl of 25 × 10^−3^ M HAuCl_4_ and 50 to 60 μl of 0.01 M AgNO_3_ sequentially, resulting in formation of bright orange color complex of [AuBr_4_]^−^. After that, 32 μl of 0.1 M ascorbic acid was added to the solution under gentle stirring and was stirred for 5 minutes. Upon addition of ascorbic acid, the solution turned to colorless, indicating the reduction of Au^3+^ to Au^1+^. At the last step, 50 μl of seed solution was added to this solution and the mixture left undisturbed overnight.

### Synthesis of PEG-modified SPIONs

As of co-precipitation is a simple popular method for synthesis of super paramagnetic iron oxide nanoparticles, we applied it with some modifications^37^. Briefly, 5 ml solution of 2 M HCl, containing 0.004 and 0.002 moles of Fe^3+^ and Fe^2+^, respectively, was prepared. Excess amounts of m-PEG and diacid-PEG (5:1) were dissolved in this solution. Then it was titrated by 50 ml of 0.7 M ammonia under stirring. It should be noted that the nanoparticle’s size distribution was highly affected by ammonia addition rate. Under dropwise addition, the mixture color was changed to black through pH increasing. The black sediment was separated by external magnetic field, washed three times with methanol and redispersed in 50 ml of methanol. This process resulted to formation of 0.4 gr SPIONs.

### Synthesis of GIHN

The synthesis of the GIHN was started with a modification of GNRs by cystamine. A 400 μl portion of 20 mM cystamine dihydrochloride was added to 10 ml of just synthesized GNRs and was gently stirred. The suspension was left undisturbed for 45 minutes. Afterwards, the GNRs solution was diluted 4 times. A 100–300 μl aliquant of PEG-modified SPIONs was diluted 10-fold and then it was added dropwise to the GNRs solution under gentle stirring in the presence of EDC and NHS. By applying this method, more than one GNR could be attached to one SPION and the hybrid nanostructure contained higher fraction of GNRs. This suspension was allowed to stand for an hour before PEG addition. In the last step, a mixture of 6 kDa m-PEG-acid and 10 kDa diacid-PEG was added to the suspension. The suspension was kept in room temperature for 1 hour. The GIHNs were separated from free SPIONs through centrifugation. Finally, the suspension was magnetically washed several times to remove CTAB and excess reagent completely. The hybrid nanoparticles were then redispersed in 10 ml DI water.

### *In vitro* cytotoxicity

Mouse fibroblast cells (L929) and MCF7 cells were purchased from Pasteur Institute of Iran and stored in liquid nitrogen. The methods were carried out in accordance with the approved Pasteur Institute guidelines with the consent of the donor. L929 cells were cultured using RPMI medium and MCF7 cells cultured using high glucose DMEM medium supplemented with 10% FBS and 1% penicillin-streptomycin solution in 75 cm^2^ cell culture flasks. Cells were cultivated in an incubator at 37 °C with 5% carbon dioxide. After reaching confluence, cells were detached, counted, and seeded in 24-well plate at 5 × 10^4^ cells per well. Cells were incubated for 24 hours which allowed cells attachment. GIHN were dispersed in fresh RPMI and DMEM mediums with designated concentrations. After the pre-incubation of cells, the culture medium was replaced with nanoparticles suspension. The cells were incubated for specified time periods and then the cell viability was measured using MTT assay standard protocol. Each test was repeated three times and the results were averaged.

### *In vitro* magnetic resonance imaging

A series of aqueous solutions containing 10, 20, 50, 100 and 200 μg ml^−1^ of GIHN was prepared for relaxivity measurements. All the measurements were conducted using 3T MRI system at room temperature. T_2_ relaxivity was determined using spin-echo acquisition utilizing 32 echo-time (TE) ranging from 12 ms to 384 ms and a repetition time (TR) of 3000 ms where the field of view was 7 cm, slice thickness was 3 mm and acquisition matrix was 256 × 128.

### APTT and PT assay

Fresh blood was drawn from healthy adult volunteer and mixed with sodium citrate as an anticoagulation agent with a volume ratio of 9:1 blood:sodium citrate. The following methods are like previous studies^45^. Platelet poor plasma (PPP) was collected by spinning the citrated whole blood at 1000 G-force for 10 min. A special amount of GIHN solution in PBS is mixed with the PPP to obtain concentration of 200 μg ml^−1^ of GIHN in the plasma. The APTT and PT of samples were measured on an automatic coagulation analyzer with addition of relevant reagents at 37 °C.

### Peripheral blood smear tests

A solution of 200 μg ml^−1^ of GIHN was prepared in the fresh blood (EDTA- anticoagulated) and was shacked continuously for 10 minutes. Then a drop of the blood sample was spread on a glass lam and after fixation by methanol at room temperature, the Geimsa stain was used to colorize the blood cells.

All the blood tests were carried out in accordance with the approved protocols of ethics committee of Imam-Khomeini Hospital and with the consent of the donor. All the experimental protocols approved by the medical laboratory of Imam-Khomeini Hospital.

It should be mentioned that for all the experiments involving human subjects (cellular and blood tests), written informed consent was obtained.

## Conclusion

GNRs were used as photothermal agents^50^,^51^ with two limitations. First, total remove of toxic CTAB molecules led to missing high percentages of GNRs^12^. The second problem is low stability of GNRs in CTAB-free media. As a modification, hybrid GNR-based nanostructures were synthesized^11^,^17^. However, decreasing the intensity of plasmon resonance absorption usually occurs after these modifications forcing use of high laser irradiation powers for photothermal therapy^23^.

As a solution for the mentioned problems, a new bi-functional GNR-based nanostructure was proposed and synthesized by a cost-effective high-yield method for photothermal therapy and MR imaging. In this process plasmo-magnetic nanostructure was obtained by mixing cystamine modified GNRs and carboxylic acid functionalized SPIONs at room temperature. The attachment of nanostructures occurred in concentrated CTAB media. In contrast to other reported processes, no centrifugation step which normally causes GNRs destruction was needed. The synthesis parameters were optimized so that the new GIHN exhibited enhanced stability with similar plasmonic behavior of bare GNRs. The biocompatibility of the nanostructure was achieved by total replacement of toxic CTAB molecules with biocompatible PEG ones. The MTT assay test results showed that even after 48 hours of incubation more than 85% of cells survived. Also the synthesized nanostructure shows excellent hemocompatibility bearing no change in the plasma coagulation and RBCs morphology. Given the small size, near IR optical properties and biocompatibility, the hybrid nanoparticles were applied as a photothermal agent and caused around 70% decrease in cell viability. Collections of superparamagnetic nanoparticles in the GIHN provide clustering effect which enhanced the MRI contrast. The synthesized plasmo-magnetic nanoparticles have free carboxylic groups on their surface which make them appropriate for drug loading. So this new nanostructure is promising for employment in a multimodal biomedical imaging as magnetic resonance imaging (MRI) contrast agent and simultaneous photothermal and chemotherapy. Further research on drug loading and photothermal therapy is in progress.

## Additional Information

**How to cite this article**: Khafaji, M. *et al*. A new bifunctional hybrid nanostructure as an active platform for photothermal therapy and MR imaging. *Sci. Rep*. **6**, 27847; doi: 10.1038/srep27847 (2016).

## Supplementary Material

Supplementary Information
