# Supplementary material for: A new bifunctional hybrid nanostructure as an active platform for photothermal therapy and MR imaging
Source: Sci Rep. 2016 Jun 14;6:27847. doi: 10.1038/srep27847 (PMC4906516; doi:10.1038/srep27847)
Supplement: Supplementary Information [file srep27847-s1.doc]

Supporting Information

**A new bifunctional hybrid nanostructure as an active platform for photothermal therapy and MR imaging**

Mona Khafaji^1^, Manouchehr Vossoughi^2,3,*^ , M. Reza Hormozi-Nezhad^4,*^ ,Rassoul Dinarvand^5^ ,Felix Börrnert^6,7^ , Azam Irajizad ^1,8^

^1^Institute for Nanoscience and Nanotechnology, Sharif University of Technology, Tehran 14588, Iran.

^2^Department of Chemical and Petroleum Engineering, Sharif University of Technology, Tehran, Iran.

^3^Institute for Biotechnology and Environment (IBE), Sharif University of Technology, Tehran, Iran.

^4^Department of Chemistry, Sharif University of Technology, Tehran 11155-9516, Iran.

^5^Nanotechnology Research Center, Faculty of Pharmacy, Tehran University of Medical Sciences, Tehran, Iran.

^6^IFW Dresden, PF 270116, 01171 Dresden, Germany.

^7^Speziallabor Triebenberg, TU Dresden, 01062 Dresden, Germany.

^8^Department of Physics, Sharif University of Technology, Tehran 14588, Iran.

^*^E-mail: [vosoughi@sharif.edu](mailto:vosoughi@sharif.edu), hormozi@sharif.edu

**
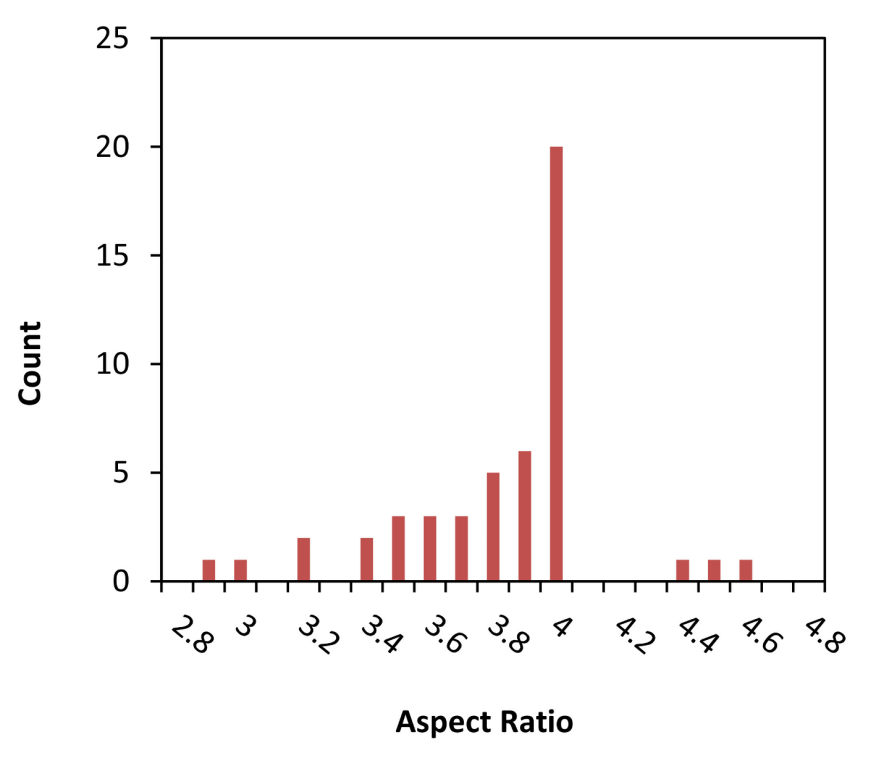
**

**Figure S 1.** Aspect ratio distribution of GNRs which obtained from TEM images.


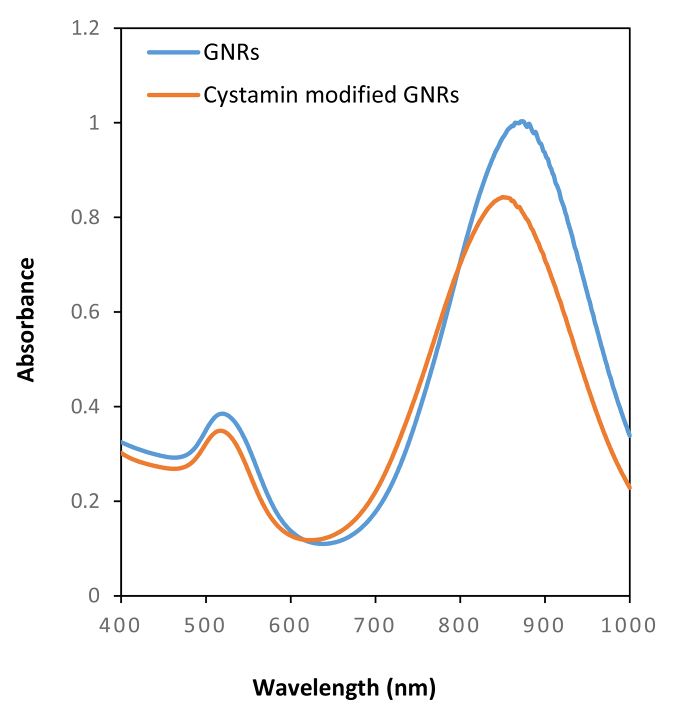


**Figure S 2.** UV-Vis absorption spectra of just synthesized GNRs (blue line) and cystamine modified GNRs (red line).


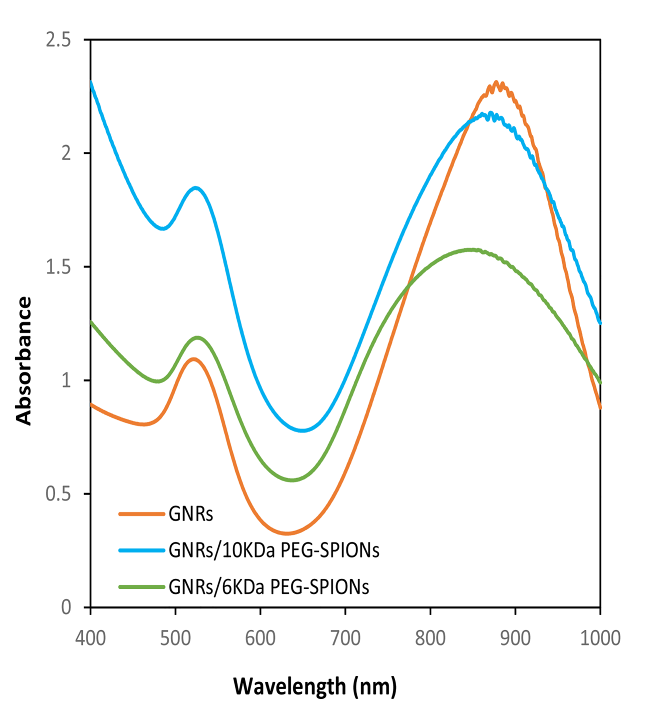


**Figure S 3.** UV-Vis spectra of GNRs (red line), GNR-10 kDa PEG modified SPIONs (blue line) and GNR- 6 kDa PEG modified SPIONs (green line).


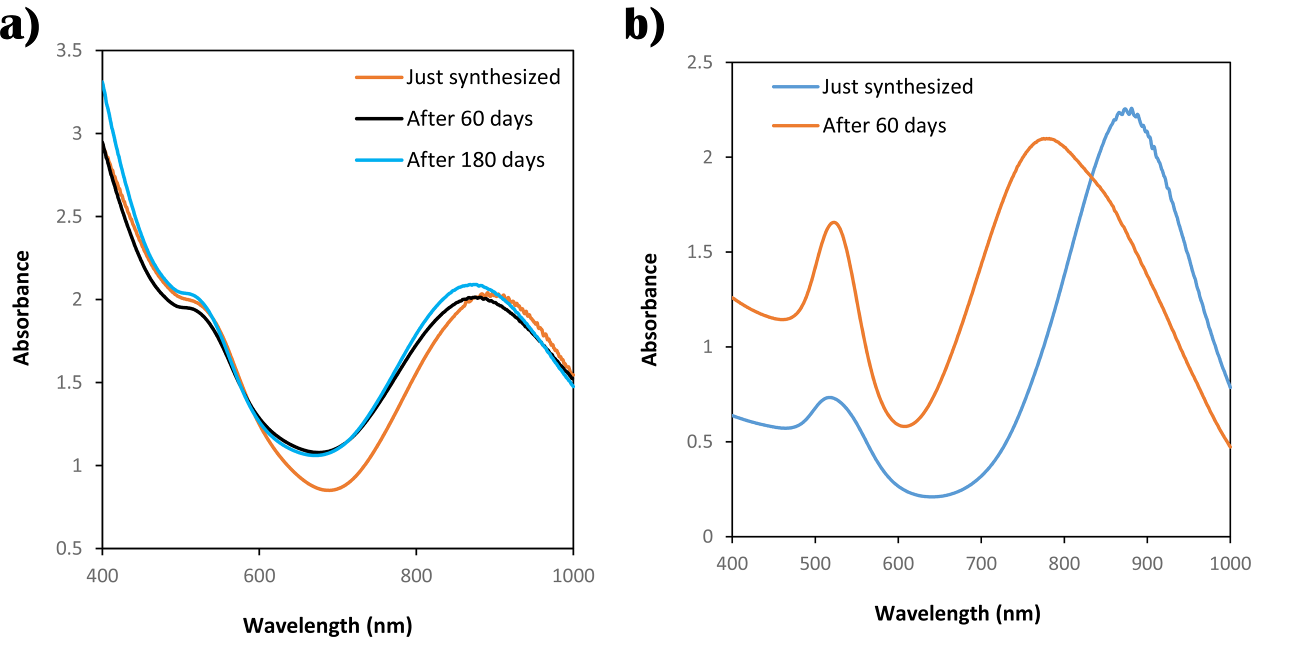


**Figure S 4.** UV-Vis spectra of GIHN suspension after synthesis (red line), after 60 days (black line) and after 180 days (blue line) (a) and UV-Vis spectra of GNRs solution after synthesis (blue line) and after 60 days (red line) (b).


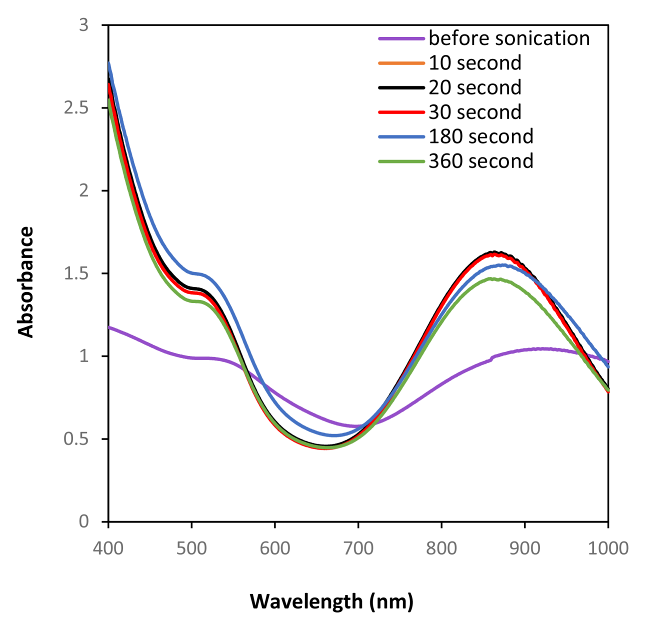


**Figure S 5.** UV-Vis spectra of GIHN suspension before sonication (purple line) and after 10 (red line), 30 (black line), 180 (blue line) and 360 (green line) seconds of sonication.


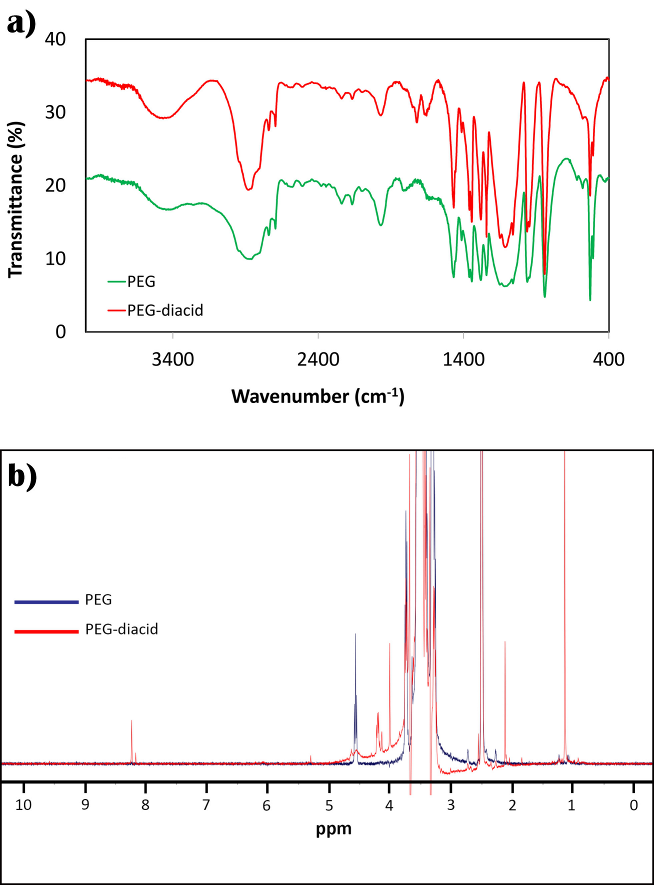


Figure S 6. FTIR spectrum of polyethylene glycol and dicarboxylic acid-PEG (a) and H^1^NMR spectrum of polyethylene glycol and PEG-diacid (b).
